# A cross-sectional study on selected child health outcomes in India: Quantifying the spatial variations and identification of the parental risk factors

**DOI:** 10.1038/s41598-020-63210-5

**Published:** 2020-04-20

**Authors:** Parul Puri, Junaid Khan, Apurba Shil, Mohammad Ali

**Affiliations:** 1 Doctoral Fellow, International Institute for Population Sciences, Govandi Station Road, Deonar, Mumbai, 400088 India; 2 Doctoral Fellow, International Institute for Population Sciences, Govandi Station Road, Mumbai, 400088 Maharashtra India; 3 International Institute for Population Sciences, Govandi Station Road, Deonar, Mumbai, 400088 India; 4 Doctoral Fellow, Department of Public Health, Faculty of Health Sciences, Ben-Gurion University of the Negev, Beersheba, Israel; 5 Senior Scientist, Department of International Health, Johns Hopkins University, Baltimore, Maryland 21205 United States of America

**Keywords:** Malnutrition, Public health, Epidemiology

## Abstract

This study examined association between selected child health indicators- anaemia, stunting and no/incomplete immunization by inter-linking maternal characteristics at district level and parental characteristics at individual level. A spatial analysis and a binary logit model estimation were employed to draw inferences using the data from the fourth round of National Family Health Survey, 2015–16 of India. Significant spatial clustering of the selected child health outcomes was observed in the country. Mother’s educational attainment explained significant district level differential in the selected child health outcomes. At the individual level, parents who are very young, not-educated, socially excluded, belong to poor class were found to be significantly associated with the poor child health outcomes. This study indicates that parental characteristics, such as age, educational attainment and employment substantially determine child health in India, suggesting that an intervention by targeting the households where children are vulnerable is important to improve child health in the country.

## Introduction

Approximately 6.3 million children under five years of age die every year, globally^1^. Nutritional deficiency is the leading cause of under-five mortality in the developing countries. On the other hand, incomplete-or-no-immunization puts the children at risk of infection from the diseases like Polio, Measles, Tuberculosis and other vaccine preventable diseases (VPDs). And, immunization against the VPDs among the children reduce the risk of mortality and morbidity associated with these deadly infectious diseases^2^. It was estimated that during 2008, seventeen percent of all the deaths among children age 0–59 months were vaccine preventable^3^. This suggests that one in every three children does not achieve their full developmental potential^3^. As part of the public health challenge, India could not achieve the targeted Millennium Development Goals (MDGs) and lagged behind in terms of the child health indicators like nutritional status and immunization^2^. Maintaining an improved child health, thus, becomes a key challenge for social and capital growth in the country^3,4^ and through the sustainable development goals India is aiming to achieve zero hunger and malnutrition by 2030.

India carries the second largest population share in the world with diverse culture and dietary habits^5^. Therefore, it is essential to incorporate these diversities into consideration while making plans and policies. Also, a major part of the country has been clinched with patriarchy and women subordination from the times immemorial^6^. This patriarchal setup imparted supremacy to the male members of the family, who possess the status of the primary meal-ticket and decision maker. Paternal influences, regarding socio-economic, demographic and lifestyle factors can, therefore, be observed on child health outcomes.

India carries a substantial burden in terms of child health, which is clear from the level of anemia, stunting, and immunization coverage in the country^7–11^. From the estimates generated by the three consecutive rounds of the National Family Health Survey (NFHS-2, 1998–99; NFHS-3, 2005–06; NFHS-4, 2015–16), a transition in the level of child health outcomes can be observed. The proportion of anaemia amongst children was 74.3 percent (1998–99)^12^, 69.5 percent (2005–06)^13^ and 58.4 percent (2015–16)^14^. Also, the proportion of full immunization amongst children was 42.0 percent (1998–99)^12^, 43.5 percent (2005–06)^13^ and 62.0 percent (2015–16)^14^. The proportion of stunted children has reduced by 15 percent from year 1998–99 (46%) to 2015–16 (38%)^14^ (Supplementary File S1). The country has achieved continued financial progress (over 5% growth in GDP) and decreased the poverty level by half (50% in 1993–94 to 22% in 2011–12). However, the improvement in the child health outcomes are still not at par with the international targets set by the MDGs^10,11^.

Existing literature suggests that nutritional status, blood anaemia level and VPDs, play a vital role in determining the overall development of child health^10,15^. Stunting can be defined as a severe form of undernutrition and is identified as the height that is below two standard deviations from the median child growth standard laid by the World Health Organization (WHO)^16^. Stunting proliferates the risk of poor health, which may affect scholastic and economic well-being of an individual in their later life^13,15^. WHO defines anemia as a condition where either the number of red blood cells or their oxygen-carrying capacity becomes insufficient to satisfy the physiological needs of an individual’s body^1^. Children suffering from anaemia have weak immunity and are therefore at higher risk of having infections. Moreover, iron deficiency which is supposed to be the major cause of anemia highly affects the cognitive capacities like learning, memory and behavior of the children throughout their lifetime^16^. Full immunization has emerged out as a worthwhile method to prevent a group of lethal diseases, generally referred to as VPDs^17^. However, a large proportion of children in India, still do not receive all the doses of the full immunization schedule (around 40%) and it remains a key public health challenge^14^.

There is abundant literature which focuses on exploring the child health outcomes in terms of socio-economic factors in different country settings. Though these studies explored the spatial heterogeneity and correlates of child malnutrition and different doses of full immunization coverage^15,17^, the relationship between child health (malnutrition) and health care utilization (immunization) with paternal characteristics has not been previously explored. Recent developments in public health research suggests that geospatial mapping and modeling of different demographic and epidemiological events by inter-linking different exposures help in the identification of the pockets and therefore, help in accurate resource allocation. This would, furthermore, help the government and policymakers to address the need of the community and, therefore, achieve the latest targets set by the Sustainable Development Goals (SDGs)-2015-30.

Thus, the present study aims to explore the existence of spatial heterogeneity in the selected child health outcomes (i.e., stunting, anaemia and no-or-partial-immunization) on the basis of parental socio-economic and demographic factors at both macro and micro-level. The study employed a district level spatial analysis prior to identifying the inter-district variances in the selected child health outcomes after addressing spatial clustering and neighbourhood effect.

## Methods

### Data source and Sampling technique

This study used the data from the fourth round of National Family Health Survey (NFHS-4), 2015–16 (https://dhsprogram.com/data/dataset/India_Standard-DHS_2015.cfm?flag=1) conducted under the stewardship of Ministry of Health & Family Welfare, Government of India with the help of International Institute for Population Sciences (IIPS) as the nodal agency^18^. All experimental protocols of the survey were approved by the nodal agency IIPS, India. The survey aimed at providing national and sub-national level estimates on population, health, and nutrition and other key demographic indicators. Informed consent was obtained from all the subjects involved in this survey. Since the data utilized in this study has been archived in a public repository, therefore, it is easily accessible and there is no need of ethical approval for conducting this study. The digital district level map of India was obtained from GitHub (https://github.com/datameet/maps/tree/master/Districts), which is shared under Creative Commons Attribution 2.5 India license. The map was re-projected in WGS 1984 UTM zone 43N for conducting spatial analysis. The study did not include parts of Jammu & Kashmir and Gujarat, as the survey estimates were not available for these areas. The sampling design adopted by NFHS-4 was a multistage stratified sampling considering urban and rural areas as the natural strata. All methods used in this study were carried out in accordance with relevant guidelines and regulations.

### Defining the sample size and the outcome variable

The study utilized a nationally representative sample of 25,563 parents (Father 15–54 years and Mother 15-49 years)-child (12–59 months) pair data for the individual level analysis. In addition, estimates for all the 640 districts were computed from the kids file of DHS (IAKR74FL) (n = 211,028) after stratifying the analysis to all those kids of age-group 12–59 months. The child health outcomes of interest were childhood anaemia, stunting and, immunization dropout (No-or-partial-immunization).

### Defining Macro- and Micro- level predictor variables

The study employed both macro and micro-level parental information on socio-economic and demographic factors as predictors. The demographic factors included in the study are age of both the parents (in years) and place of residence (rural/urban). All the variables included in the study are selected in accordance with the framework proposed by the Commission on Social Determinants of Health (CSDH) set up by the WHO. The framework establishes the socio-economic context as the structural determinant of health-related outcomes. The framework further suggests that these socio-economic context can be indicated by factors like gender, social group, religion, level of education, occupation and wealth. The framework further emphasizes that these structural determinants influences the intermediary determinants such as, material circumstances, behavioural, biological and psychological factors which in turn interferes with the inequality in health and well-being of any individual^19^.

It is to be noted that NFHS does not provide any information on income or expenditure, therefore, the present study utilizes Demographic and Health Surveys (DHS) wealth index to measure the socio-economic status of the respondent. A DHS wealth index is computed using the information available on assets and amenities present in a household. A detailed description on calculation of DHS wealth Index can be found from DHS comparative report^20^. Table 1 provides a detailed variable description.

**Table 1:** Description of the variables included in the study.

District level variables	
Outcome variables	Description
Childhood anemia (%)	Proportion of children aged 12–59 months who were anaemic (<11.0 g/dl) in the district
Childhood stunting (%)	Proportion of children aged 12–59 months who were stunted (height-for-age) in the district
No and Partial childhood immunization (%)	Proportion of children aged 12–59 months and who were not fully immunized (BCG, measles, and 3 doses each of Polio and DPT) in the district
**Independent variables**	**Description**
Mother of Age 15–24 years (%)	Proportion of mothers aged 15–24 years in the district
Mother Uneducated (%)	Proportion of uneducated mothers in the district
Mother Unemployed (%)	Proportion of unemployed mothers in the district
Rural (%)	Proportion of mothers living in rural areas
Poor (%)	Proportion of mothers having poor economic status
Non-Hindu (%)	Proportion of mothers from non-Hindu communities
Scheduled Castes/Tribes (%)	Proportion of mothers from Scheduled Caste (SC)/Scheduled Tribe (ST) communities
**Individual Level Variables**	
Outcome variables	Description
Childhood Anaemia	Non-Anaemic = 0, Anaemic = 1
Childhood Stunting	Non-Stunted = 0, Stunted = 1
Childhood Immunization	Full Immunization = 0, Partially or Not immunized = 1
Independent variables	Description
Parental Age	Both Young (Both the parents aged between 15–24 years)
	Only Mother Young (Mother aged between 15–24 years)
	Only Father Young (Father aged between 15–24 years)
	Both Older (Father aged 25–54 years & Mother aged 25–49 years)
Residence	Urban /Rural
Parental Education	Both not educated
	Only father completed at least primary education
	Only mother completed at least primary education
	Both father and mother completed at least primary education
Parental Occupation	Both unemployed
	Only father working
	Only mother working
	Both are working
Caste	Scheduled Castes (SC)/ Scheduled Tribes (ST)
	Other Backward Class (OBC)
	Non-SC/ST and non-OBC
Religion	Hindu/Muslim/Non-Hindu and Non-Muslim
Wealth	Poorest/Poorer/Middle/Richer/Richest
Region	Northern(Chandigarh, Delhi, Haryana, Himanchal Pradesh, Jammu and Kashmir, Punjab, Rajasthan and Uttrakhand)
	North-eastern (Arunachal Pradesh, Assam, Manipur, Meghalaya, Mizoram, Nagaland, Sikkim, and Tripura)
	Central (Chhattisgarh, Madhya Pradesh, Uttar Pradesh)
	Eastern (Bihar, Jharkhand, Odisha, West Bengal)
	Western (Dadra and Nagar Haveli, Daman and Diu, Goa, Gujrat and Maharashtra)
	Southern (Andaman and Nicobar Islands, Andhra Pradesh, Karnataka, Kerala, Lakshadweep, Puducherry, Tamil Nadu, and Telangana)

India has a hierarchical social stratification structure which was historically built on the basis of the occupation performed by various groups. For the present analysis, the social group (caste) has been categorised into three categories, namely Scheduled Castes/Tribes (SC/ST), Other Backward Class (OBC) and Non-SC/ST-and-non-OBC. Scheduled Castes/Tribes are the historically most disadvantaged social groups, whereas OBC falls in between the traditional upper caste and the lowest social groups^21^.

### Analytical approach

Unweighted frequency and weighted percentage distribution were computed to describe the sample under consideration. District-level quantile maps were generated to understand the spatial variation of the selected child health outcomes in the country. To examine geographical clustering of child health outcomes, univariate local Moran’s *I* and Local indicator of Spatial Association (LISA) cluster maps were created. We created the spatial weight matrix (*w*) of order one using the Queen’s contiguity method (neighbors sharing a common boundary of non-zero length) to quantify the spatial proximity between each possible pair of observational entities^17^.

Moran’s *I* takes values in between −1 to +1, and a positive spatial autocorrelation indicates that points with similar attribute values are closely distributed in space whereas negative spatial autocorrelation indicates that closely associated points are more dissimilar. A zero indicates a random spatial pattern with no spatial auto-correlation. Univariate LISA measures the correlation of neighborhood values around a specific spatial location^22,23^. It determines the extent of spatial randomness and clustering present in the data^23^.

Four types of spatial auto-correlation were generated based on the four quadrants of Moran’s scatter plots, namely hotspots (districts with high values, with similar neighbors), cold spots (districts with low values, with similar neighbors), and spatial outliers (districts with high values, but with low-value neighbors and vice-versa). The cluster map is a special choropleth map showing those locations with a significant local Moran’s *I* statistic classified by the type of spatial auto-correlation where red color represents the hot spots, green represents the cold spots, and light blue and light red color represents the spatial outliers^22^.

To explain the global relations between selected child health indicators and the independent variables under study we applied Ordinary Least Squares (OLS) regression initially. Then we proceeded to build the spatial models. The OLS model was diagnosed to check the spatial dependence in the error terms by the corresponding Moran’s I value of the residuals of the OLS model. A statistically significant Moran’s I neccessarily implies application of geo-spatial models to develop and to get the unbiased estimates of the associations.

With a prior assesment of the neighbourhood effect in the outcome variables we chose to build the spatial autoregressive models- Spatial Lag Model (SLM) and Spatial Error Model (SEM). Thus, we employed the spatial models to correct the spatial bias and to get the refined estimates. Additionally, the diagnostics of the spatial dependence gave the statistical significance of Lagrange Multiplier (LM) and Robust Lagrange Multiplier for both the models for each of the outcomes. Since LM-lag, LM-error and Robust LM-lag were significant for anaemia (but Robust LM-error was insignificant); we used the results of the spatial lag model (SLM) as final for anaemia and took the results of the spatial error model (SEM) to evaluate sensitivity of the estimates obtained from the spatial lag model. A similar pattern was observed for no-or-partial-immunization also, but here Robust LM-lag was insignificant, thus we used spatial error model (SEM) as final model and utilized the results of SLM to evaluate SEM model estimates sensitivity. However, in case of stunting, we found similar pattern like anaemia and retained with the SLM for this outcome also and utilized the estimates of SEM to evaluate the sensitivity of the fitted SLM in this case. Supplementary File S2 provides the Moran’s I values in the residuals from the spatial models.

Additionally, to examine the association between the micro-level parental factors and the concerned child health outcomes in the country, we applied a multivariate logistic regression analysis. To get the adjusted estimates we included children’s socio-economic and demographic information as covariates in the analyses.

Stata version 12.0 (StataCorp^TM^, Texas) was used for the micro level analysis; Arc-GIS version 10.1, (Esri, California), R Studio version 1.2.1335 (2009–2019 RStudio, Inc) and Geo-Da version 1.12.1.129, (Teknowledgist, New York) were used for the macro (district) level analysis. All the estimates provided in this study are derived by applying appropriate sampling weights supplied by National Family Health Survey (NFHS-4), 2015–16.

## Results

### Macro-level differentials of child health: Spatial Pattern of child health outcomes across 640 districts in India

**Figure 1:**
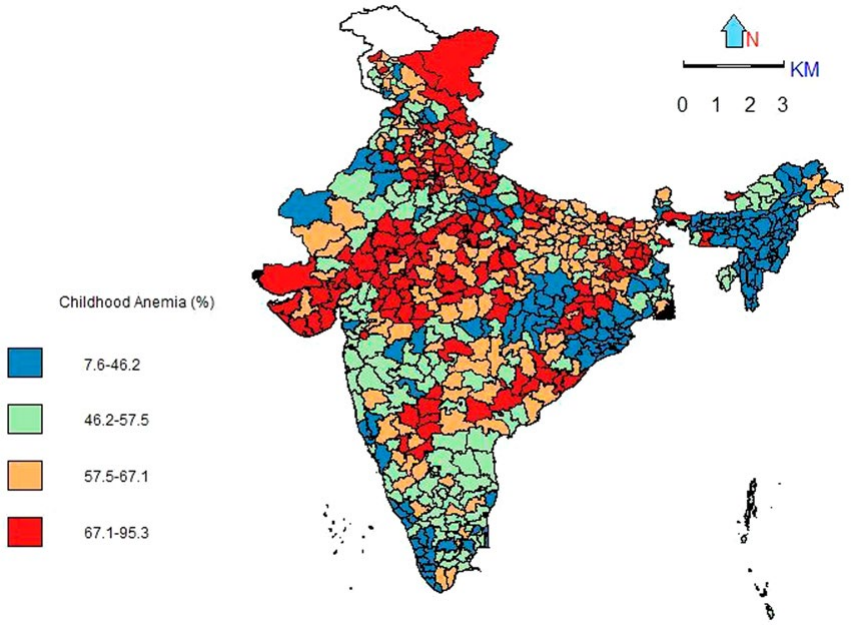
Spatial pattern of anaemia among children aged 12–59 months, India, 2015–16.

**Figure 2:**
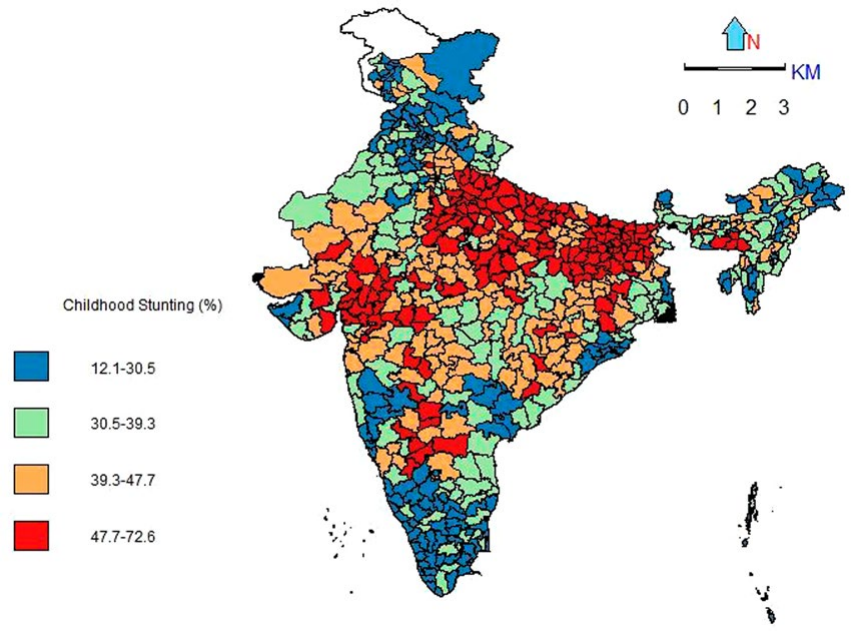
Spatial pattern of stunting among children aged 12–59 months, India, 2015–16.

**Figure 3:**
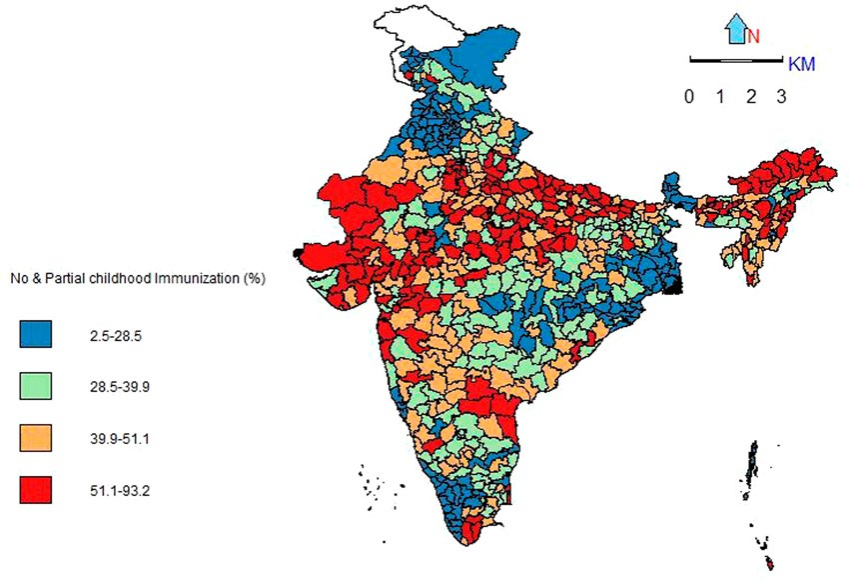
Spatial pattern of no or partial immunization among children aged 12–59 months, India, 2015–16.

Figures 1–3 depict spatial patterns of the selected child health outcomes across 640 districts of the country. The figures depict the presence of geographical heterogeneity among the selected child health outcomes in the country. It is worth mentioning, that the North-South heterogeneity remains existing and eclectic in terms of the child health outcomes.

The spatial pattern of childhood anaemia shows the occurrence of high prevalence of anaemia is concentrated in the Central, Northern, Western, and Eastern parts of the country. The result shows that 196 districts have a high prevalence (more than 65%) of childhood anaemia.

The spatial pattern of childhood stunting shows existence of the sub-national inequalities across districts of the country. The occurrence of childhood stunting is very high among most of the districts from the states like Uttar Pradesh (UP), Madhya Pradesh (MP), Rajasthan, Bihar, and, Jharkhand. On the contrary, prevalence is lower among Southern states, Jammu and Kashmir, Maharashtra, Goa and some of the North-Eastern states.

More than 318 districts in the country have over 40 percent no-or-partially-immunized child population mostly from the North-Eastern states, Gujarat, Rajasthan, Uttar Pradesh and Madhya Pradesh. However, the prevalence of no/partial immunization was found to be lower among the states of Punjab, West Bengal and Kerala.

### Spatial clustering of child health outcomes in India

**Figure 4:**
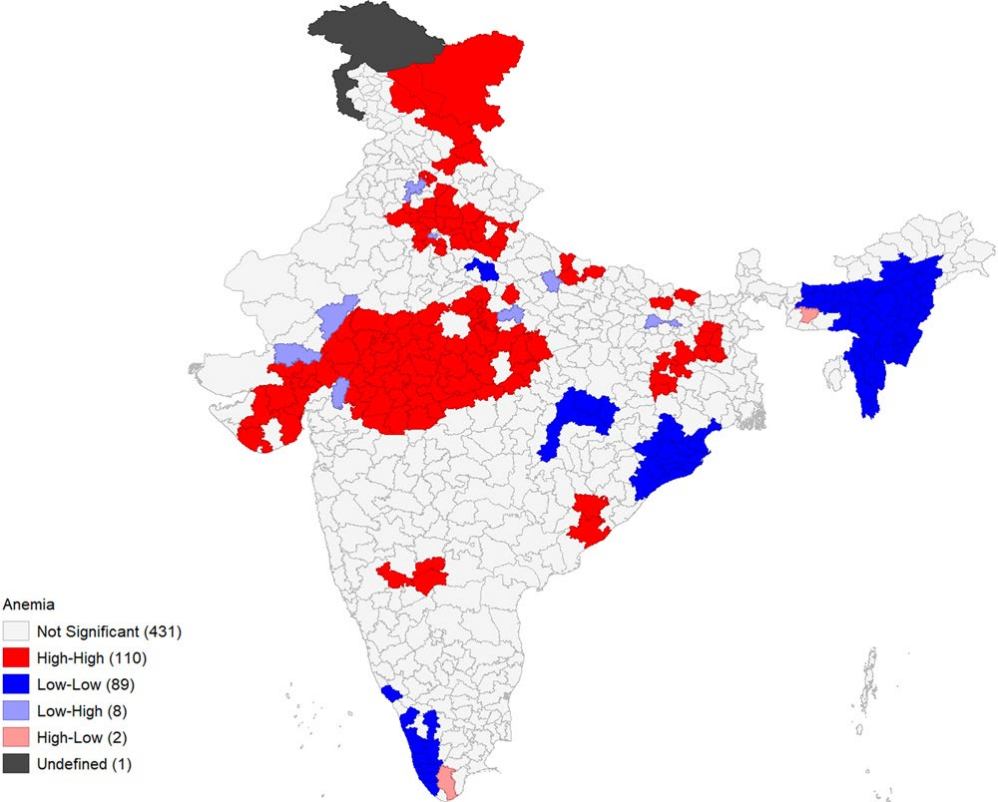
Univariate LISA Clustering of anaemia prevalence among children aged 12–59 months.

**Figure 5:**
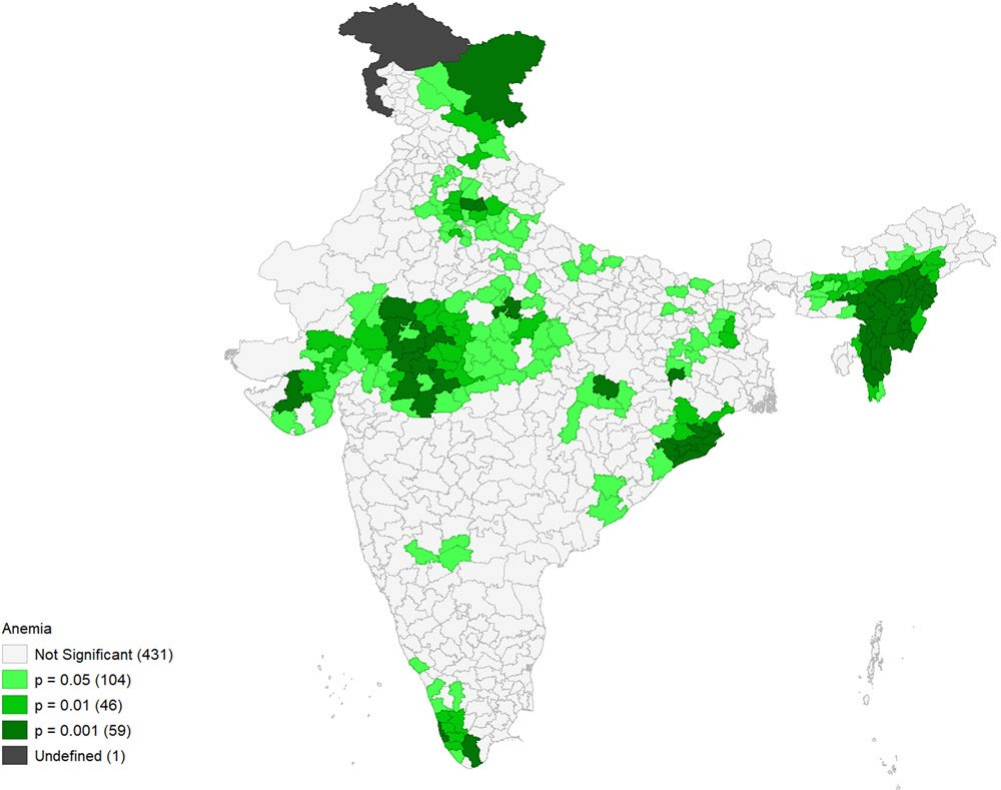
Univariate LISA significance map of anaemia prevalence (Moran’s I = 0.63***).

**Figure 6:**
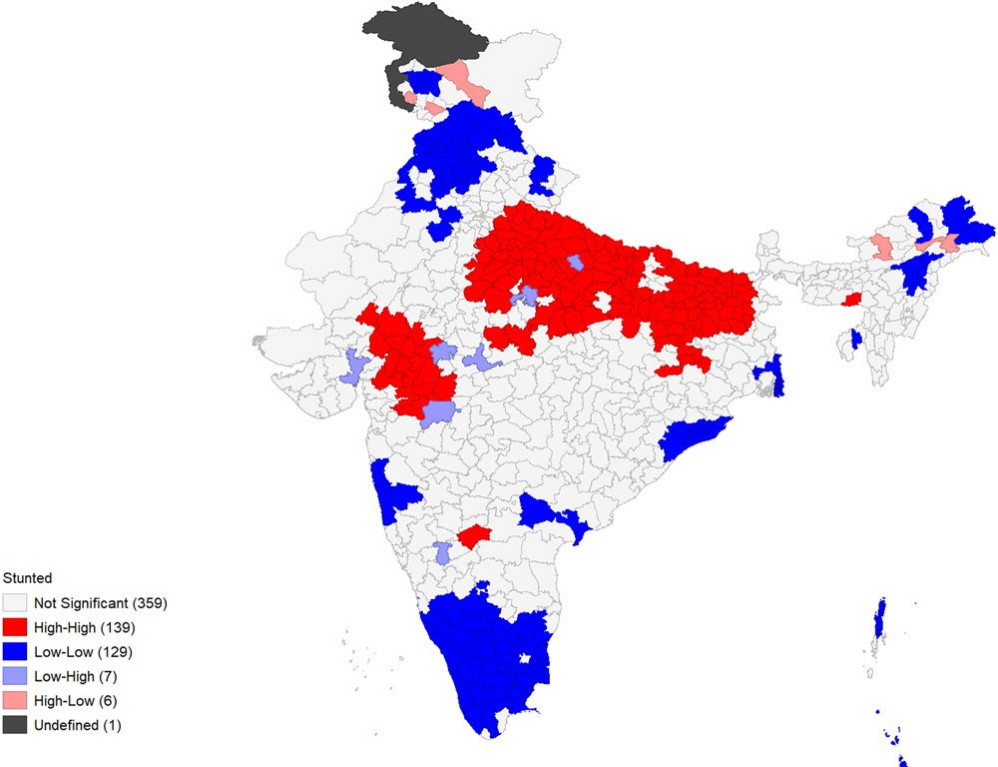
Univariate LISA Clustering of stunting prevalence among children aged 12–59 months.

**Figure 7:**
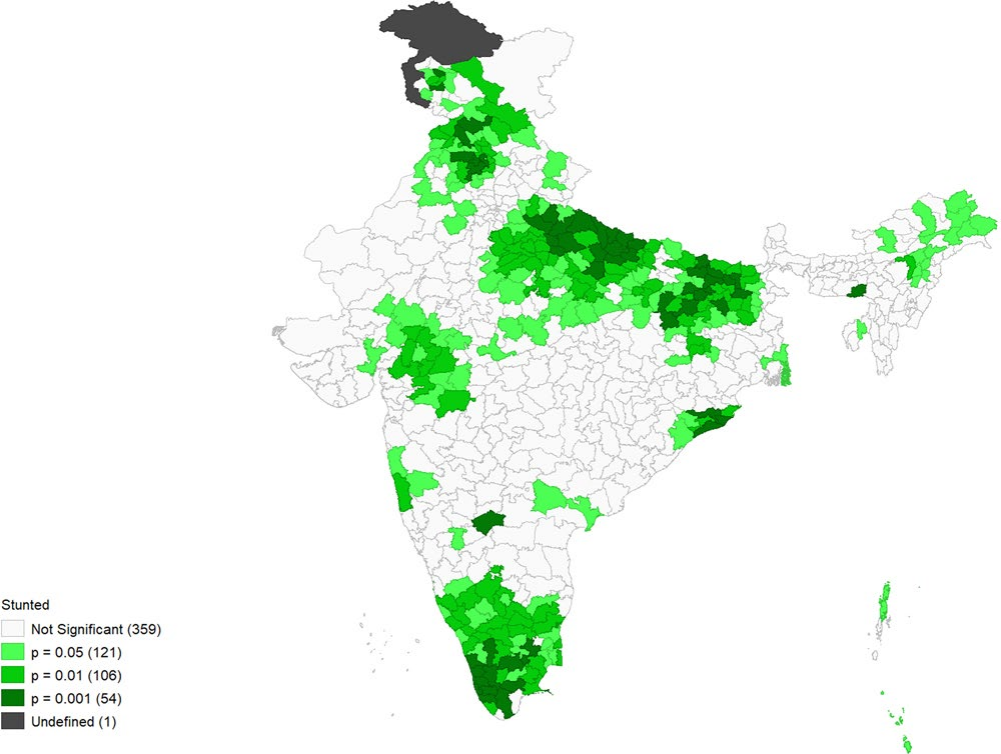
Univariate LISA significance map of stunting prevalence (Moran’s I = 0.68***).

**Figure 8:**
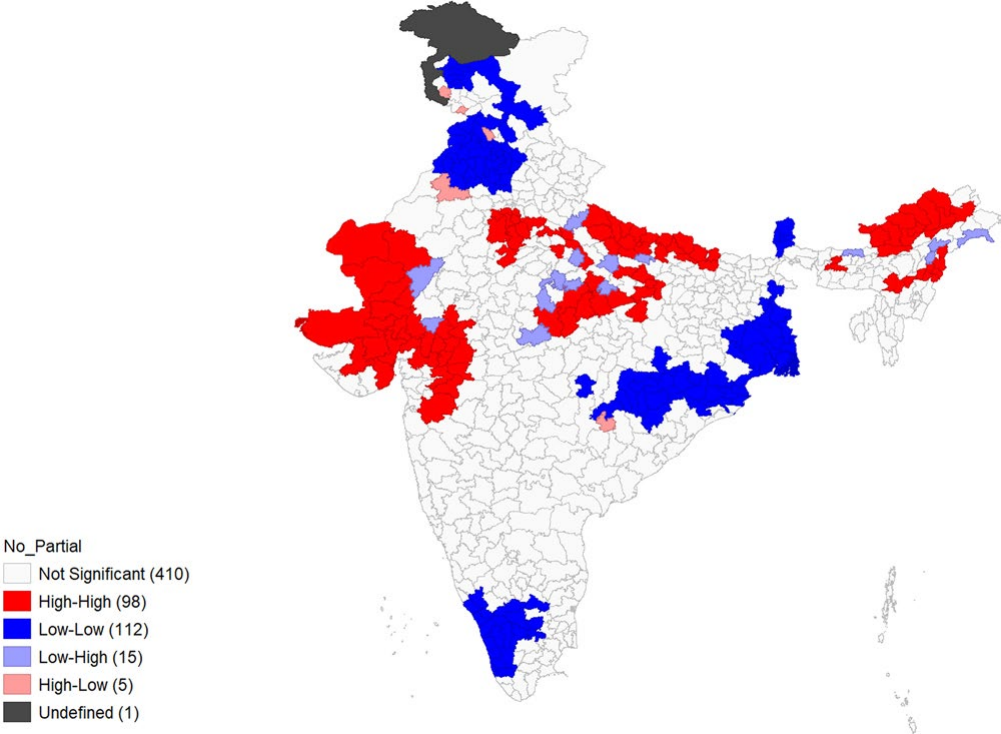
Univariate LISA Clustering of no or partial immunization among children aged 12–59 months.

**Figure 9:**
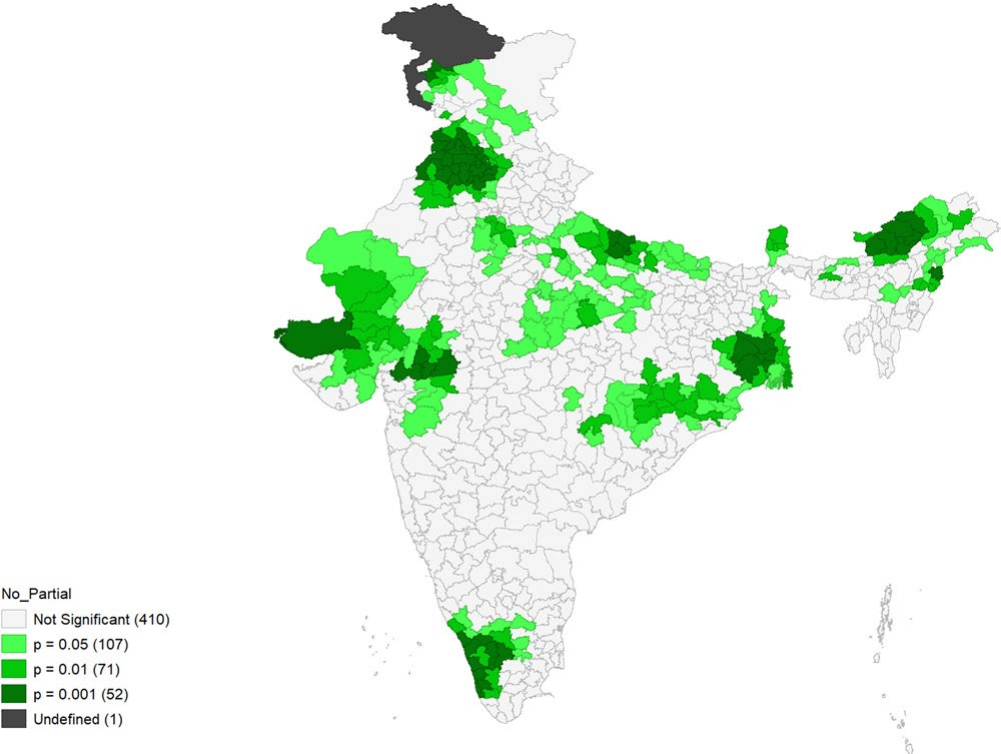
Univariate LISA significance map of no or partial immunization (Moran’s I = 0.61***). ‘***’ is significant at 1% level of significance.

Figures 4–9 show the measures of spatial clustering of child health outcomes across districts. All three outcomes of child health show a considerably higher spatial auto-correlation. Spatial clustering is highest for childhood stunting (Moran’s *I* = 0.68, p-value = 0.001).

Univariate LISA cluster map of childhood anaemia (Moran’s *I* = 0.63, p-value = 0.000) identified 110 ‘hotspots’ and 89 ‘cold spots’. Most of the hotspots are located in Madhya Pradesh (MP) and parts of Uttar Pradesh (UP), Gujarat, Jharkhand and Bihar (Fig. 4). Whereas, it is lower in the states of Kerala, Odisha, and North-Eastern states like Nagaland, Assam, Tripura, and Manipur.

The LISA cluster map for childhood stunting generated 139 ‘hotspots’ and 129 ‘cold spots’ across the districts of India. The hotspots were mostly located in Uttar Pradesh, Bihar and parts of Gujarat and Jharkhand.

Significant spatial clustering (Moran’s *I* = 0.61, p-value=0.001) was also observed for childhood no-or-partial-immunization, where 98 ‘hotspots’ and 112 ‘cold-spots’ were identified. The hotspots were mostly situated in Rajasthan, Uttar Pradesh and parts of the North-eastern states, Gujarat, some parts of Maharashtra and Madhya Pradesh (MP).

### Spatial modeling of child health outcomes

Previous section explored the geographical differences in the selected child health outcomes across districts, i.e. the second administrative and policy relevant units within states (first administrative unit) in India. This section also hinted the presence of spatial heterogeneity in the selected child health outcomes with statistically significant autocorrelation. Taking spatial dependency in the selected child health outcomes, into account, the study shows the estimated spatial models, regressing on the covariates previously determined by the study framework. We primarily built up the spatially weighted OLS model and examined the Moran’s I value of the error component for each of the outcome variables. We found Moran’s I value of the error to be statistically significant for each of the health outcomes, which confirmed spatial endogeneity in the OLS model (Supplementary File S3).

Table 2 presents the estimates of the spatial lag models (SLM) for anaemia and stunting and Spatial Error Model (SEM) for no-or-partial-childhood-immunization. The values of the coefficients showed the level of association between the child health outcomes and district level maternal characteristics. Estimated coefficients for anaemia suggested that district level proportion of mothers having no education, low poverty and mothers belonging to non-Hindu population were highly associated with district level prevalence of anaemia among children (aged 12–59 months). Empirically, a 10-point increase in the percentage of no educated mothers across the districts was associated with 2.3 unit increase in the anaemia prevalence. While showing a negative association, the corresponding beta coefficients suggested that with one unit increase in the proportion of poor and Non-Hindu population across the districts were associated with 0.09 and 0.04-point reduction in the anaemia prevalence respectively. This meant that districts with higher percentage of not educated mothers carried the higher burden of child anaemia whereas districts with higher share of poor and non-Hindus in the total population carried the lower burden of childhood anaemia. A negative association between district level percentage of poor population and prevalence of child anaemia indicates a comparative pro-rich distribution of child anaemia burden across the districts. Though poor population (the lowest two quintiles of wealth index measure) carries the burden of anaemia largely but the counter population of the district (rest three non-poor quintiles of wealth index) shares a larger share of the child anaemia burden in the population within and across the districts. Though it needs a careful exploration of the unit level data to measure the likelihood of being anaemic for a child from those households in the lowest two quintiles compared the rest of the well-off quintiles.

**Table 2:** Spatial lag and error estimation of child health outcomes (Anaemia; Stunting; No or Partial immunization) across 640 districts, India, 2015–16.

Predictors	Anaemia (Spatial Lag model output)			Stunting (Spatial Lag model output)			No or Partial immunization (Spatial Error model output)		
	Coefficient	S.E.	p-value	Coefficient	S.E.	p-value	Coefficient	S.E.	p-value
Mother of Age 15–24 (%)	0.05	0.05	0.245	0.05	0.03	0.104	0.12	0.07	0.098
Mother Uneducated (%)	0.23	0.03	0.000	0.15	0.02	0.000	0.26	0.05	0.000
Mother Unemployed (%)	−0.04	0.03	0.232	−0.06	0.02	0.001	−0.03	0.04	0.425
Rural (%)	−0.01	0.02	0.734	0.003	0.01	0.821	0.004	0.03	0.898
Poor (%)	−0.09	0.02	0.000	0.08	0.02	0.000	0.07	0.05	0.103
Non-Hindu (%)	−0.04	0.02	0.021	0.001	0.01	0.883	0.11	0.03	0.000
Scheduled Caste/Tribe(s) (%)	0.02	0.02	0.397	−0.05	0.01	0.000	0.005	0.03	0.853
Lag (Rho)/Error (Lambda) Coefficient	Lag (Rho) Coefficient.= 0.61			Lag (Rho) Coefficient.= 0.57			Error (Lambda) Coefficient = 0.74		

Mother’s education played a strong statistical association with each of the district level prevalence of child health outcomes. Predominantly, anaemia and rate of no-or-partial-immunization across the districts showed higher association with educational pattern of the mothers. This association appeared to be comparatively lesser in case of stunting prevalence. Other than education and unemployment status across districts, proportion of poor, Scheduled Caste/Tribe(s) population in the districts were observed to be statistically associated with stunting prevalence. It was found that an increase (10 point) in the poor population share can actually increase the stunting prevalence by 0.8 unit whereas a 10 point increase in the percentage of Scheduled Caste/Scheduled Tribe (s) population across the districts had shown a decrease in the stunting prevalence. Here in case of the stunting model, we observed that once adjusted for other district level characteristics, proportion of mothers unemployed across the districts showed a negative association with child stunting prevalence which indicates that increase in the percentage of unemployed mothers across the districts is associated with decrease in stunting prevalence. This indicates that the district level prevalence of stunting among those children of employed mothers is comparatively higher and predominant than their counter part across India’s districts. Though employment for an individual is directly associated with the source of income and in India mothers choose to work outside on sheer economic necessity^24^ though in Indian context and due to its patriarchal structure of the society, mothers don’t go for outside work and are mostly unemployed which directly allows the mother to look after and rearing up their children for a longer duration of the day. On the contrary, studies show that mother’s employment has an adverse effect on child health^25^. It is true that household’s food consumption directly depends upon the level of income and in a family both the parents may get engaged in some kinds of employment and can contribute to the household’s total income. But most commonly in India, only the father of the household is engaged in employment and earns for the family. Thus the mother, eventually unemployed, look after their children in the household. In this context, this study further explores the data at unit level beyond the district level and the corresponding hypothesis on parent’s employment status is examined in the next section. But the district level analyses apparently makes it clear that districts with higher percentage of unemployed mothers showed the lower burden of child stunting across the districts.

The model estimates of no-or-partial-immunization suggested that maternal education and non-Hindu religious status across the districts played a statistically significant role to predict the district level prevalence of no-or-partial immunization among the children aged 12–59 months. The lag and error coefficients from the SLM and SEM estimation for anaemia, stunting and no-or-partial immunization were found to be 0.61, 0.57 and 0.74 respectively. The values of the lag and error coefficients represented the spatial diffusion process referring to the common shocks in the spatial events. Here in our study we restricted ourselves from estimating the spatial diffusion matrix as we already assumed the child health outcomes to be dependent upon the variables within the study framework. The aforesaid result findings clearly depicted that mothers’ demographic status especially educational attainment and poverty level across the districts are substantially associated with child health indicators.

Supplementary File S4 gives the estimates from the SEM and SLM for the sensitivity check. However, SEM estimation for stunting was redundant and should not be referred due to the nature of the spatial dependency of the indicator. The SEM and SLM confirmed the sensitivity in the results suggesting any presence of the unobserved factors in the model equation may substantially predict the nature of the association between the health outcomes and the independent variables conceptualised within the study framework.

### Description of the study population for the unit level analysis

The study is based on the 25,563 parents-child pair from the most recent round of data from the National Family Health Survey (NFHS), 2015–16.

Table 3 provides a description of the eligible parents in age group 15–54 years (fathers) and 15–49 years (mothers). The majority (72%) of the study sample comprised of couples, where both the partners were in older-age groups. The rural-urban distribution of the sample shows that 26 percent of the eligible parents were from the urban areas. Of the total sample 65 percent of the parents completed at least primary education. And among the 70 percent of the parents, only father was found to be working in the family. Around 39 percent of the eligible parents belonged to Scheduled Caste/Tribes (SC/STs) and another 38 percent belonged to Other Backward Class (OBC) category. About 72 percent of the population belonged to the religion Hindu. Additionally, more than 47 percent of the eligible parents were from poor wealth quintile and had relatively low-economic well-being. The regional distribution shows nearly 29 percent of the samples came from the Central part of India followed by the Northern region (18%), Eastern region (18%), North-eastern region (14.43%), Southern region (12%), and Western region (9%).

**Table 3:** Description of the variables included in the unit level study.

Child health Outcome variables	Sample Size (N)	Proportion (%)
**Anaemia**		
Non-Anaemic	10,599	41.46
Anaemic	13,926	54.48
Missing	1,038	4.06
**Stunting**		
No	13,965	54.63
Yes	9,951	38.93
Missing	1,647	6.44
**Immunization status**		
Fully immunized	15,596	61.01
No/partially immunized	9,967	38.99
**Background Characteristics**		
**Parental Age**		
Both Young	1,536	6.01
Mother Young	5,406	21.15
Father Young	126	0.49
Both Older	18,495	72.35
**Parental Education**		
Both Not Educated	3,150	12.32
Only Father completed at least Primary	4,487	17.55
Only Mother completed at least Primary	1,214	4.75
Both of them completed at least Primary	16,712	65.38
**Parental Occupation**		
Both Unemployed	1,321	5.17
Only Father Working	17,751	69.44
Only Mother Working	357	1.40
Both Working	6,134	24.00
**Residence**		
Urban	6,647	26.00
Rural	18,916	74.00
**Caste**		
Scheduled Caste (SC)/Scheduled Tribe (ST)	10,096	39.49
Other Backward Classes (OBC)	9,702	37.95
Non-SC/ST and Non-OBC (Ref.)	5,765	22.55
**Religion**		
Hindu	18,387	71.93
Muslim	4,087	15.99
Others	3,089	12.08
**Wealth**		
Poorest	6,126	23.96
Poorer	5,888	23.03
Middle	5,346	20.91
Richer	4,399	17.21
Richest	3,804	14.88
**Region**		
Northern	4,716	18.45
North-eastern	3,690	14.43
Central	7,302	28.56
Eastern	4,500	17.60
Western	2,394	9.37
Southern	2,961	11.58
**Total**	25,563	

### Micro-level predictors of child health outcomes

Table 4 presents the bivariate distribution of selected child health outcomes, namely childhood anaemia, childhood stunting, and no-or-partial Immunization. Further in Table 5, Model 1 shows that variables like parental age, education, caste, wealth, religion and region were found to be significantly associated with childhood anaemia. The results depict that the children of couples, where both father and mother belonged to the young age-group (15–24 years), both not educated, belonging to Muslim, Scheduled Caste/Tribe(s) category, belonging to poorest economic status, hailing from Northern, Central, and Western region of the Country had higher likelihood of having childhood anaemia (p-value < 0.05).

**Table 4:** Prevalence (per 100 children) of the selected child (aged 12–59 months) health outcomes (N = 25,563) by background characteristics, India, 2015–16.

Background variables	Anaemia	Stunting	No/Partial immunization
**Parental Age**	χ^2^ p-value = 0.000	χ^2^ p-value = 0.000	χ^2^ p-value=0.000
Both Young	68.21	50.28	40.72
Mother Young	58.38	40.23	33.71
Father Young	53.86	33.70	34.68
Both Older	55.70	40.79	39.74
**Parental Education**	χ^2^ p-value = 0.000	χ^2^ p-value = 0.000	χ^2^ p-value = 0.000
Both Not Educated	64.83	57.28	53.81
Only Father completed at least Primary	62.40	53.62	49.26
Only Mother completed at least Primary	61.93	51.08	38.36
Both of them completed at least Primary	54.06	34.64	32.93
**Parental Occupation**	χ^2^ p-value = 0.888	χ^2^ p-value = 0.000	χ^2^ p-value = 0.000
Both Unemployed	57.64	40.14	45.09
Only Father Working	56.49	39.83	37.12
Only Mother Working	59.77	46.88	43.68
Both Working	58.69	45.37	40.15
**Residence**	χ^2^ p-value = 0.000	χ^2^ p-value = 0.000	χ^2^ p-value = 0.000
Urban	54.40	32.25	35.55
Rural	58.26	45.10	39.54
**Caste**	χ^2^ p-value = 0.000	χ^2^ p-value = 0.000	χ^2^ p-value = 0.000
Scheduled Castes/Tribes (SC/ST)	60.06	48.13	39.43
Other Backward Classes (OBC)	57.19	40.88	39.19
Non-SC/ST and Non-OBC	52.95	32.59	35.28
**Religion**	χ^2^ p-value = 0.000	χ^2^ p-value = 0.000	χ^2^ p-value = 0.000
Hindu	57.12	41.33	37.98
Muslim	59.67	42.40	43.48
Others	48.18	34.98	26.77
**Wealth**	χ^2^ p-value = 0.000	χ^2^ p-value = 0.000	χ^2^ p-value = 0.000
Poorest	63.54	56.18	48.79
Poorer	58.79	48.82	41.57
Middle	57.67	39.95	35.26
Richer	53.72	31.66	33.01
Richest	48.90	22.85	29.87
**Region**	χ^2^ p-value = 0.000	χ^2^ p-value = 0.000	χ^2^ p-value = 0.000
Northern	62.80	37.04	34.30
North-eastern	36.08	36.42	45.39
Central	63.14	49.80	46.51
Eastern	56.39	43.30	30.38
Western	55.50	39.81	46.10
Southern	51.48	32.28	32.00
India	57.09	41.20	38.31

**Table 5:** Adjusted odds ratio (AOR) of having adverse child health outcomes by the parental characteristics in India, NFHS-4, 2015–16.

Background variables	Anaemia (Model 1)	Stunting (Model 2)	No/Partial-Immunization (Model 3)
	AOR with 95% C.I	AOR with 95% C.I	AOR with 95% C.I
**Parental Age**			
Both Young (Ref.)	1.00	1.00	1.00
Mother Young	0.79***(0.70–0.90)	0.91 (0.81–1.03)	0.81*** (0.72–0.92)
Father Young	0.61***(0.41–0.89)	0.80 (0.54–1.19)	0.86 (0.58–1.25)
Both Older	0.66***(0.59–0.74)	0.81*** (0.73–0.91)	0.89* (0.80–0.99)
**Parental Education**			
Both Not Educated(Ref.)	1.00	1.00	1.00
Only Father completed at least Primary	0.89* (0.81–0.98)	0.95 (0.87–1.05)	0.90* (0.82–0.99)
Only Mother completed at least Primary	0.94 (0.82–1.09)	1.02 (0.88–1.17)	0.67*** (0.58–0.77)
Both of them completed at least Primary	0.78*** (0.71–0.85)	0.70***(0.640.77)	0.53***(0.49–0.58)
**Parental Occupation**			
Both Unemployed (Ref.)	1.00	1.00	1.00
Only Father Working	0.99 (0.88–1.12)	1.06 (0.94–1.20)	0.73*** (0.65–0.82)
Only Mother Working	0.92 (0.72–1.18)	1.24 (0.96–1.59)	0.90 (0.70–1.14)
Both Working	1.01 (0.89–1.14)	1.07 (0.94–1.22)	0.67***(0.59–0.76)
**Residence**			
Urban	1.03 (096–1.10)	1.02 (0.95–1.10)	1.05 (0.98–1.13)
Rural (Ref.)	1.00	1.00	1.00
**Caste**			
Scheduled Caste/Tribe (SC/ST)	1.17*** (1.08–1.26)	1.34*** (1.23–1.45)	1.24*** (1.14–1.34)
Other Backward Classes (OBC)	1.09* (1.10–1.17)	1.27*** (1.17–1.37)	1.26***(1.17–1.36)
Non-SC/ST and Non-OBC (Ref.)	1.00	1.00	1.00
**Religion**			
Hindu	1.10 (0.99–1.21)	0.94(0.84–1.04)	1.02 (0.92–1.13)
Muslim	1.25*** (1.10–1.41)	1.09 (0.96–1.23)	1.28***(1.13–1.45)
Others (Ref.)	1.00	1.00	1.00
**Wealth**			
Poorest	1.46*** (1.30–1.63)	2.94*** (2.61–3.32)	2.05*** (1.83–2.30)
Poorer	1.33*** (1.20–1.47)	2.48*** (2.23–2.77)	1.55*** (1.40–1.72)
Middle	1.28*** (1.16–1.41)	1.85*** (1.67–2.06)	1.20*** (1.09–1.33)
Richer	1.10 (1.00–1.21)	1.37*** (1.23–1.52)	1.15*** (1.04–1.27)
Richest (Ref.)	1.00	1.00	1.00
Region			
Northern	1.51*** (1.36–1.66)	1.07(0.96–1.19)	0.90 (0.81–1.00)
North-eastern	0.42*** (0.37–0.47)	0.86* (0.76–0.98)	1.80***(1.60–2.02)
Central	1.35*** (1.23–1.48)	1.45*** (1.32–1.60)	1.34*** (1.22–1.48)
Eastern	1.04 (0.94–1.16)	1.11(0.99–1.23)	0.63*** (0.57–0.71)
Western	1.18** (1.05–1.32)	1.43*** (0.26–1.61)	1.74*** (1.55–1.95)
Southern (Ref.)	1.00	1.00	1.00

**Note**. *p < 0.05, **p < 0.01, ***p < 0.001.

In Model 2, parental age, education, caste, religion, wealth and region of residence were found to be significantly associated with childhood stunting (p-value < 0.05). Furthermore, the children whose both parents had no education, belonged to Scheduled Caste/Tribe(s) and OBC category, belonging to poorest economic status and reside in the Central, Western, and North-eastern region of the country had higher likelihood of suffering with childhood stunting.

Findings from Model 3, show that children whose parents belong to young age-group, have no formal education, unemployed, belong to Scheduled Castes/Tribes and Other Backward Class (OBC), belong to Muslim faith, belong to poor wealth quintile and hail from the North-Eastern, Western and Central part of India have the lower chance of being fully vaccinated.

To demonstrate the precision of statistical estimations being done at the unit level, estimated standard errors of the selected indicators are provided in the Supplementary File S5.

## Discussion

The present study exhibits the evidences on large disparity in receiving all the doses of basic immunization and the public health burden in terms of child stunting and anaemia among the study group of children by their background characteristics, parental characteristics in particular and across districts. Spatial analysis captures the neighbourhood effect and informs about the district level variation in the burden of the selected child health indicators. Whereas, the unit level analysis is the intended in-depth analysis to identify the micro-level predictors of the child health outcomes beyond the macro-level framework

Beyond the district level spatial analyses, the novelty of this study is that it is based upon the parent-child pair data compiled from the National Family Health Survey (NFHS), 2015–16 dataset. Though we could not aggregate the parent-child pair data at the district level due to sample size issue, we performed the district level analysis where we could only utilize the mother’s information and her child/children. As the study extends to the unit level, we examined the child health disparities in terms of the parental characteristics more specifically subject to the other covariates within the conceptual framework of the study. Previous studies corroborate the maternal factors and its linkages with child health outcomes in different country settings. However, Indian society is profoundly influenced by the paternal factors in the household decision making, and therefore, the decisions concerning child health are based on the prejudice, socio-economic well-being and educational status of the father^8^. Thus, it becomes essential to re-conceptualize the child health scenario in India through the lens of both maternal and paternal characteristics. The present study aims to draw attention towards the combined effect of the parental background characteristics, which substantially predict the selected child health outcomes. In the dataset, father’s information is only available in the state module and not at the district level. Therefore, the district-level spatial analysis is restricted to mother-child pair. The extended unit level analysis tags father’s information with the existing mother-child information from the corresponding datasets of NFHS, 2015–16.

The district level spatial analysis suggested that there exists geographical variation in the child health outcomes, like, anaemia, stunting and no-or-partial-immunization among the children under-five years of age across the districts of India. District level neighbourhood effect plays a crucial role to affect child health outcomes across India^15,26^. The findings on the level of childhood anaemia are well aligned with the existing literature, which is higher for the states of Uttar Pradesh, Madhya Pradesh (Central region), and parts of Bihar and Jharkhand (Eastern region) of the country^11^. Stunting was found to be higher for the Central (state of Uttar Pradesh), Eastern (Bihar) region and parts of the state Jharkhand and Gujarat. These findings are similar to that indicated by the report published by *‘POSHAN Abhiyaan*’, Ministry of Women and Child Development, Government of India^27^. The report also highlights that the states mostly from Central India have an alarmingly high level of Stunting^10^. Partial-or-no-immunization was found to be higher for the Central (Uttar Pradesh and Madhya Pradesh), Northern (Rajasthan) and North-eastern region respectively^27^. These results corroborate with the existing literatures which suggest that Northern (Rajasthan), Central (Madhya Pradesh, Uttar Pradesh), North-eastern, and Western (Gujarat, some parts of Maharashtra) region of the country still have a lower prevalence of full immunization^17,28,29^.

Apparently it is evident that Central, Eastern, Northern and North-eastern region of the country are lagging behind in one or more selected child health outcomes. This may be owed to the poor health infrastructure in India^30,31^. The existence of socio-economic inequality in access to health care persists in India and is mentioned in the studies bygone^30,31^. These inadequacies in the health infrastructure exists because of the huge area, population size and, density in the Northern and Central regions of the country^32^. Moreover, the accessibility and utilisation of the healthcare facility is hampered by the malpractices and corruption which predominate the existing healthcare infrastructure in the country^29,33^. Additionally, the healthcare system of North-eastern states is considerably more fragile as compared to other parts of the country, which may be attributed to the extensive variations in terms of terrain and uneven population density throughout the region^32–34^.

The district level spatial modelling of the aggregated data shows that districts with higher proportion of young mothers aged in between 15–24 years carry higher burden of child stunting (β = 0.05; p-value = 0.104) and no-or-partial immunization (β = 0.12; p-value = 0.098). Mother’s educational attainment is significantly associated with all the three selected child health outcomes and higher percentage of no-educated mothers across the districts is associated with higher burden of anaemia, stunting and no-or-partial immunization among the children in those districts. The problem of malnutrition increases where the mothers are unaware of the nutritional value of the food that their children consume, and therefore, the level of all the three child health outcomes are poor for the children whose mothers have no education^10,35–40^. The district level aggregated data shows no statistically significant association between mother’s unemployment and anaemia prevalence and percentage of children who did not complete the doses of full immunization except stunting prevalence. Notably, the association between mother’s unemployment and stunting prevalence is negative which indicates that districts with comparatively higher proportion of mothers unemployed carry a lower burden of child stunting. The findings also show that anaemia and stunting are affected by wealth, and thus, children of the poor mothers are at higher risk of having adverse health outcomes across the districts. Wealth is a key decisive factor for the dietary practices that an individual follows^41^. Consequently, it becomes obvious that individuals from lower wealth quintile are less likely to eat a nutritious meal. Additionally, the food supplies provided by the existing public distribution system (PDS) is not enough to mitigate the burden of child malnutrition in India^42^.

Additionally, there is evidence that no-or-partial immunization is more common among the children whose mother belong to non-Hindu religion. Additionally, childhood stunting was found to be higher amongst the children whose mother belonged to Scheduled Castes/Tribes (SC/STs). In case of religion and caste no existing evidence was found. However, in India the dietary practices, occupational activities, and attitudes^35^ are often monitored by the religion and caste of a person, and thus may have influence on the selected child health outcomes.

The intended unit level analysis brings forth the association between parental characteristics-age, education and employment with the child health indictors. From the unit level analysis, it emerges that parental age (young), education, occupation, religion, caste, and wealth significantly determines child health parameters in India. It is noteworthy that the children whose parents are young in age, not educated, belong to social group SC/ST, OBC and Muslim, and those who belong to poor wealth quintile are more prone to suffer from all the three selected child health outcomes^30,37,43^.

This study projects the age (parents’) association of child health indicators-stunting, anaemia. The district level analysis confirms the association (significant at 10%) between mother’s age and higher burden of child stunting and lower coverage of full immunization. In this direction extending the analysis at the unit level, when age of both the parents is considered, we find substantial association between age of the parents and child health parameters. The likelihood is quite low for a child to be anaemic when his/her parents are older. Similarly, the likelihood is observed to be low for stunting as well as for no/partial immunization among those children whose parents are older (aged 25 years or more) compared to the children of younger parents. Previous study linked early age pregnancy and the risk of being malnourished among the children born to the adolescent mothers^44^. Adolescence pregnancy, and mother’s own nutritional status during pregnancy and during her life course plays an important role in the birth outcome and substantially determines birth weight of the new born^44^. In this direction, the evidence show that the  children of the young mothers/parents are vulnerable to be at the risk of poor nutritional health-stunted or anaemic. Though there are additional pathways through the socio-economic characteristics, that the children of the younger parents are disadvantaged against and bear the higher burden of the selected child health indicators. Thus, the effect of the parent’s age is not independent of the other socio-economic characteristics. As stunting is a chronic condition, the children are exposed to long term malnourishment until identified and intervened specially with nutritional care and health care.

It is also evident that parental education substantially predicts child health parameters. And children whose parents do not have any formal education show the higher burden of stunting, anaemia and incompletion of full immunization doses. Children from those households where both parents are formally educated (completed at least primary schooling) show the lowest likelihood for no/partial immunization followed by stunting and anaemia. In a previous study it has been shown that parent’s education and income predicts childhood anaemia. Though proper dietary  consumption and regular intake of iron can substantially reduce the burden of anaemia^43,45^, still a majority of the children under age five years are anaemic in India. Thus, it could be mentioned that educated mother/parents do take care of dietary intake and are more aware of their child’s nutritional health than those parents having no formal education^36,39,46^. Studies conducted in different country settings like- Asia, Africa, Latin America, and the Middle East show positive association between parental education and child’s nutritional status^47^ and in this study also a parental education gradient in stunting and anaemia is observed which indicates lower burden of child stunting and anaemia among the children of higher educated parents. In a different study it has been shown that education level and occupation of the mother and the father is associated with the knowledge of childhood vaccination^17^. This study also reinforces the fact that educated parents are aware about the childhood vaccination of the full immunization doses and children of the educated parents do not miss the doses compared to those children whose parents do not have any formal education.

The unit level analysis does not show any significant association between parental occupation and anaemia or stunting prevalence except no-or-partial immunization. The likelihood of missing any of the doses or not receiving any of the doses is quite low among those children whose both parents are engaged in occupation. The possible explanation could be that parents who are having employment are supposed to be educated and aware about the childhood vaccination programs and thus utilized the health care facilities to vaccinate their children.

The study supports the proposition of parental influences on the health of a child, which by far has been overlooked. Child health programs in India excluded the importance of men’s participation in the community-level awareness programs, which have been extensively designed for the eligible women population in India. Thus, the main decision makers of the household are incapable of understanding the significance of appropriate nutrition and vaccination for their children. Additionally, government led food security and distribution schemes should take into account the nutritional value of the food items stored and distributed. Despite enormous resources apportioned by the government to improve child health, India still lags in achieving the Sustainable Development Goals (SDG-2015). Maintaining an acceptable level of child health is a challenge for social and capital growth in India. An increased focus should be laid upon the geographical pockets identified in the study, i.e. Central, Northern, North-eastern and Eastern parts of the country. A fresh attention should be laid up on the existing health infrastructure in these areas. This study contributes to the understanding of the relationship between child health and parental characteristics of age, educational attainment and occupation using the household survey data from the fourth round of the National Family Health Survey. There is a potential dearth of studies in the domain of child health and the associated nexus with parental characteristics in Indian setting. In this direction, few previous studies showed the linkages between mother’s characteristics and child health but so far no study explored the parental characteristics and its influence on child health.

## Conclusion

This study is a comprehensive effort to understand the district level analogy in the differential of selected child health outcomes, crucial to understand the overall health of the child. Additionally, this study examined the association between poor child health parameters (anaemia and stunting) and vaccination care service utilization at micro-level within a cross-sectional setting. Key findings of the study aim to inform the policy makers and researchers in the field of public health research demonstrating the nexus between parental characteristics with child health and health care utilizations. And this study recommends that parents (the primary caregivers) should be well informed about their child’s nutritional health and the importance of vaccinating their children against all the basic doses of full immunization. Additionally, special care should be taken to closely monitor those households where parents are not well educated, do not have any employment or source of income and young parents who are not experienced in parenting and child care^46,47^.

## Supplementary information

Supplementary Information.
