# Supplementary material for: A cross-sectional study on selected child health outcomes in India: Quantifying the spatial variations and identification of the parental risk factors
Source: Sci Rep. 2020 Apr 20;10:6645. doi: 10.1038/s41598-020-63210-5 (PMC7170851; doi:10.1038/s41598-020-63210-5)
Supplement: Supplementary file 1 — Supplementary Information. [file 41598_2020_63210_MOESM1_ESM.pdf]

Title

A cross-sectional study on selected child health outcomes in India: Quantifying the spatial variations and identification of the parental risk factors

Author's Name

Parul Puri[1], Junaid Khan[2*], Apurba Shil[3, 4], Mohammad Ali[5]

Authors' Affiliation

[1]Doctoral Fellow,
International Institute for Population Sciences,
Govandi Station Road, Deonar,
Mumbai-400088, India
E-mail address: parulpuri93@gmail.com

[2] Doctoral Fellow,
International Institute for Population Sciences,
Govandi Station Road, Mumbai-400088,
Maharashtra, India
E-mail address: statjun@gmail.com

[3] International Institute for Population Sciences,
Govandi Station Road, Deonar,
Mumbai-400088, India
E-mail address: apurba.shil316@hotmail.com

[4]Doctoral Fellow,
Dept. of Public Health, Faculty of Health Sciences,
Ben-Gurion University of the Negev, Beersheba, Israel
E-mail address: apurba@post.bgu.ac.il

[5] Senior Scientist,
Department of International Health,
Johns Hopkins University,
Baltimore, Maryland-21205,
United States of America
Email Id: mali25@jhu.edu

*Corresponding Author

Doctoral Fellow,
International Institute for Population Sciences,
Govandi Station Road, Mumbai-400088,
Maharashtra, India
E-mail address: statjun@gmail.com
Mobile Contact: +91-9653601397

SUPPLEMANTARY INFORMATION FILE

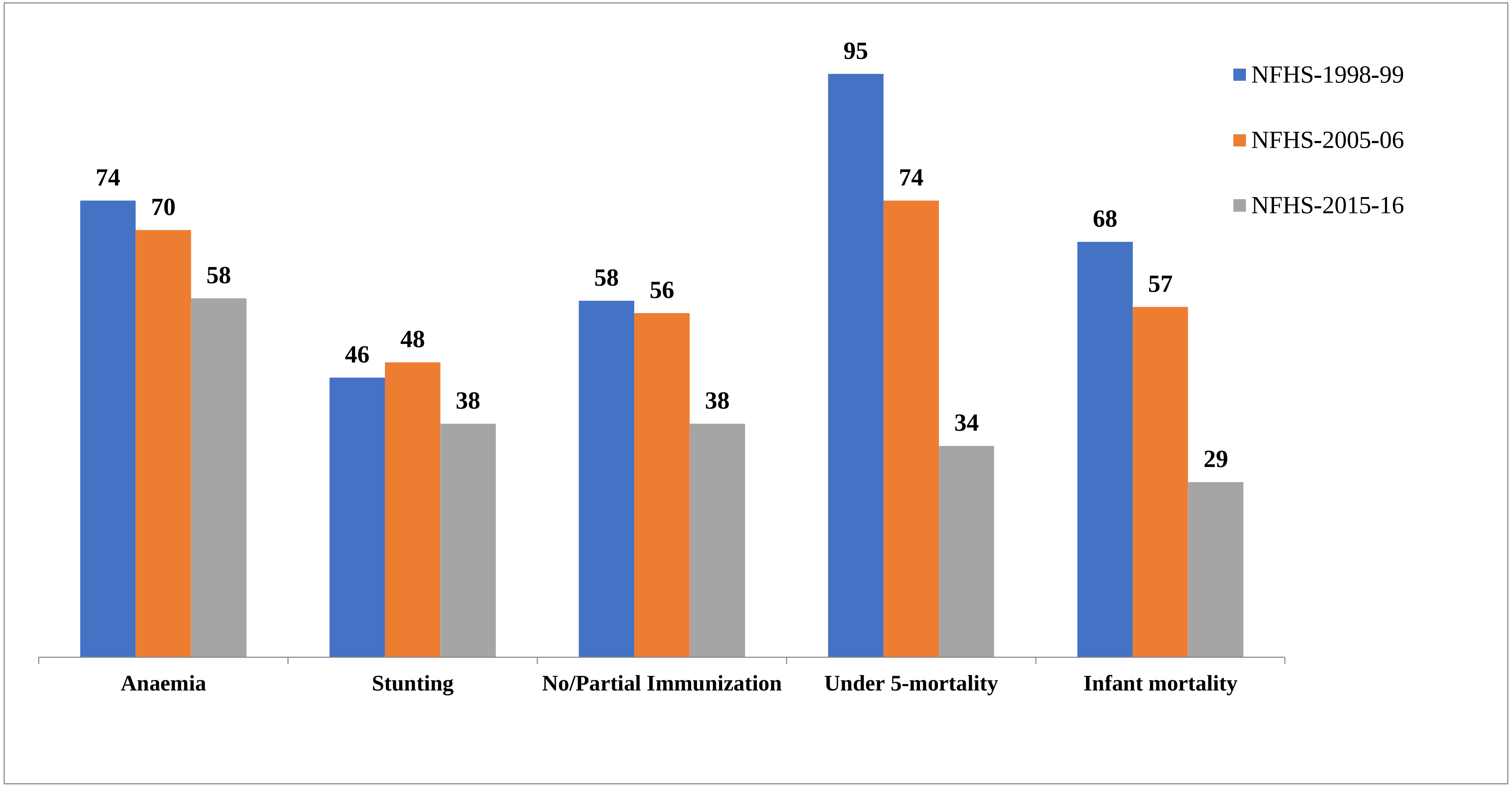

S1. Level of Child Health and mortality Indicators, India, 1998-2016

……

Outcome Variables	Spatial Models	Moran's I for residuals	p-value
Anemia	Spatial Lag	-0.066	0.002
	Spatial Error	-0.066	0.003
Stunting	Spatial Lag	0.014	0.269
	Spatial Error	-0.033	0.108
No or Partial Immunization	Spatial Lag	-0.01	0.385
	Spatial Error	-0.039	0.070

S2. Spatial Lag Model (SLM) and Spatial Error Model (SEM) model diagnostics

……

Diagnostic Tests	Anaemia		Stunting		No or Partial Immunization	
	Value	Prob.	Value	Prob.	Value	Prob.
Moran’s I (error)	16.62	0.00000	15.99	0.00000	22.20	0.00000
Lagrange Multiplier (lag)	312.64	0.00000	250.59	0.00000	438.54	0.00000
Robust LM (lag)	54.68	0.00000	32.30	0.00000	3.64	0.05648
Lagrange Multiplier (error)	257.98	0.00000	238.48	0.00000	464.60	0.00000
Robust LM (error)	0.019	0.89133	20.18	0.00001	29.70	0.00000
Lagrange Multiplier (SARMA)	312.65	0.00000	270.78	0.00000	468.24	0.00000

S3. Ordinary Least Squares model diagnostics.

……

Predictors	Anaemia (Spatial Error model output)			Stunting (Spatial Error model output)			No or Partial immunization (Spatial Lag model output)		
	Coef.	S.E.	p-value	Coef.	S.E.	p-value	Coef.	S.E.	p-value
Mother of Age 15-24 (%)	0.04	0.07	0.558	0.07	0.04	0.076	0.09	0.05	0.091
Mother Uneducated (%)	0.26	0.05	0.000	0.18	0.03	0.000	0.17	0.03	0.000
Mother Unemployed (%)	-0.02	0.03	0.508	-0.04	0.02	0.034	-0.06	0.03	0.070
Rural (%)	0.01	0.03	0.704	0.004	0.02	0.797	0.01	0.03	0.756
Poor (%)	-0.08	0.04	0.053	0.14	0.02	0.000	0.02	0.03	0.531
Non-Hindu (%)	-0.02	0.03	0.383	0.01	0.02	0.443	0.04	0.02	0.019
Scheduled Caste/Tribe(s) (%)	0.02	0.03	0.530	-0.05	0.02	0.001	0.01	0.02	0.794
Lag (Rho)/Error (Lambda) Coefficient	Lag (Rho) Coefficient= 0.73			Lag (Rho) Coefficient = 0.67			Error (Lambda) Coefficient =0.70		

S4. Sensitivity check: Spatial Error and lag estimation of child health outcomes (Anaemia; Stunting; No or Partial immunization) across 640 districts, NFHS-4, India, 2015-16.

………

Proportion	Mean (95% C.I.)	S.E.	Observation
Anemia	0.571 (0.565-0.577)	0.0032	24525
Stunted	0.412 (0.406-0.418)	0.0032	23916
No or Partial Immunization	0.383 (0.377-0.389)	0.0030	25563
Parents both Young	0.059 (0.056-0.062)	0.0015	25563
Parents both not educated	0.116 (0.112-0.120)	0.0020	25563
Parents both unemployed	0.052 (0.049-0.055)	0.0014	25563
Rural	0.693 (0.687-0.698)	0.0029	25563
Scheduled Castes/Tribes (SC/ST)	0.315 (0.309-0.321)	0.0029	25563
Hindu	0.797 (0.219-0.229)	0.0025	25563
Poorest	0.224 (0.219-0.229)	0.0026	25563
Northern Region	0.124 (0.120-0.128)	0.0021	25563

S5. Estimated standard errors of the selected indicators for the study population, India, 2015-16.

………
